# Simultaneous Determination of Procainamide and *N*-acetylprocainamide in Rat Plasma by Ultra-High-Pressure Liquid Chromatography Coupled with a Diode Array Detector and Its Application to a Pharmacokinetic Study in Rats

**DOI:** 10.3390/pharmaceutics10020041

**Published:** 2018-03-30

**Authors:** Anusha Balla, Kwan Hyung Cho, Yu Chul Kim, Han-Joo Maeng

**Affiliations:** 1 College of Pharmacy, Gachon University, Incheon 21936, Korea; aanushaballa@gmail.com; 2 College of Pharmacy, Inje University, Gimhae 50834, Korea; chokh@inje.ac.kr; 3 Department of Pharmaceutical Engineering, Inje University, Gimhae 50834, Korea

**Keywords:** procainamide, *N*-acetylprocainamide, ultra-high-pressure liquid chromatography, rat, plasma, pharmacokinetics

## Abstract

A simple, sensitive, and reliable reversed-phase, Ultra-High-Pressure Liquid Chromatography (UHPLC) coupled with a Diode Array Detector (DAD) method for the simultaneous determination of Procainamide (PA) and its major metabolite, *N*-acetylprocainamide (NAPA), in rat plasma was developed and validated. A simple deproteinization method with methanol was applied to the rat plasma samples, which were analyzed using UHPLC equipped with DAD at 280 nm, and a Synergi™ 4 µm polar, reversed-phase column using 1% acetic acid (pH 5.5) and methanol (76:24, *v*/*v*) as eluent in isocratic mode at a flow rate 0.2 mL/min. The method showed good linearity (*r*^2^ > 0.998) over the concentration range of 20–100,000 and 20–10,000 ng/mL for PA and NAPA, respectively. Intra- and inter-day accuracies ranged from 97.7 to 110.9%, and precision was <10.5% for PA and 99.7 to 109.2 and <10.5%, respectively, for NAPA. The lower limit of quantification was 20 ng/mL for both compounds. This is the first report of the UHPLC-DAD bioanalytical method for simultaneous measurement of PA and NAPA. The most obvious advantage of this method over previously reported HPLC methods is that it requires small sample and injection volumes, with a straightforward, one-step sample preparation. It overcomes the limitations of previous methods, which use large sample volume and complex sample preparation. The devised method was successfully applied to the quantification of PA and NAPA after an intravenous bolus administration of 10 mg/kg procainamide hydrochloride to rats.

## 1. Introduction

**Figure 1:**
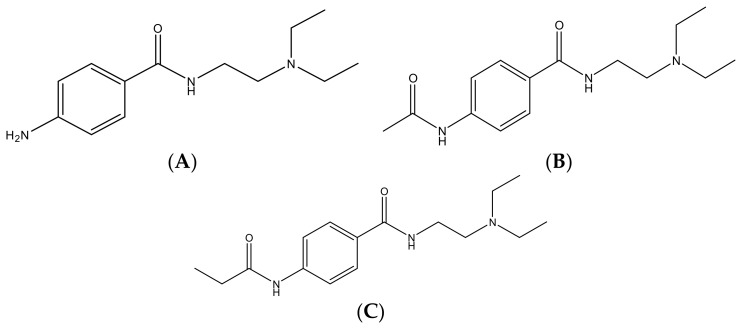
Chemical structures of (**A**) procainamide; (**B**) *N*-acetylprocainamide; and (**C**) *N*-propionylprocainamide (the Internal Standard, IS).

Procainamide (PA, *p*-amino-*N*-[2-(diethylamino)ethyl]benzamide monohydrochloride; 4-amino-*N*-[2-(diethylamino)ethyl]benzamide) (Figure 1A) is a type IA cardiac antiarrhythmic drug, which has been widely used to treat supraventricular or ventricular arrhythmia for more than 60 years [1,2], and is the drug of choice for the treatment of hemodynamically-tolerated, sustained, monomorphic ventricular tachycardia [3,4,5]. In addition to the above-mentioned therapeutic aspects, it has been shown that PA has other pharmacological effects, for example, it reduces the hepatotoxic and nephrotoxic effects of cisplatin and has been reported to have anti-inflammatory effects in a rat model of sepsis [6,7]. However, PA has potentially-serious adverse effects, such as, hypotension, polymorphous ventricular tachycardia, lupus-like syndrome, or agranulocytosis, and its applicability is limited by its narrow therapeutic window [2,8,9].

PA is predominantly metabolized to *N*-acetylprocainamide (NAPA) (Figure 1B) by *N*-acetyltransferase II in liver, and NAPA has similar pharmacologic activity to PA [10,11]. The metabolic activity for PA to NAPA shows high inter-subject variation due to a genetic polymorphism in *N*-acetyltransferase II. It has been shown that the systemic exposure ratio of NAPA/PA in rapid acetylators is higher than that of slow acetylators [10,11,12]. Because NAPA is an active metabolite, the pharmacological and adverse effects observed after PA administration might be due to both PA and NAPA. For these reasons, the determination of the concentrations of PA and NAPA in the systemic circulation is likely to be necessary to investigate the Pharmacokinetic/Pharmacodynamic (PK/PD) and/or Toxicokinetic/Toxicodynamic (TK/TD) properties, and the development of an analytical method for simultaneous determination of PA and NAPA levels in plasma is required.

A small number of methods has been devised to quantitate PA and NAPA simultaneously in plasma by Thin Layer Chromatography (TLC) [13,14], Gas-Liquid Chromatography (GLC) [15], and HPLC [16,17,18,19,20,21,22,23,24,25,26], but most lack sensitivity and/or specificity. Although several UV-HPLC methods achieved sensitivity and specificity improvements, they require multistep sample preparations that involve Liquid–Liquid Extraction (LLE), evaporation, and reconstitution. In addition, large sample volumes (0.2–2.5 mL) and injection volumes (10–100 µL) are required [16,17,18,19,20,21,22,23,24,25,26] (Table S1). To our knowledge, no method for the simultaneous determination of PA and NAPA by Ultra-High-Pressure Liquid Chromatography (UHPLC) has been described to focus on the analytical application of preclinical studies. UHPLC systems utilize small particle sizes in columns, which increases separation efficiencies and leads to better resolutions and sensitivities, and reduces the times required for analysis [27,28]. In the present study, a UHPLC-Diode Array Detector (DAD)-based method requiring simple protein precipitation for sample preparation was developed and validated for the simultaneous determinations of PA and NAPA in plasma, and successfully applied to a pharmacokinetic study in rats.

## 2. Materials and Methods

### 2.1. Chemicals and Reagents

Procainamide hydrochloride (HCl) (*M*_W_ 271.79 g/mol, purity ≥ 98.0%), NAPA (*M*_W_ 277.36 g/mol, purity ≥ 99.0%) and *N*-propionylprocainamide (NPPA, *M*_W_ 291.39 g/mol, purity ≥ 99.0% and the Internal Standard (IS) used) (Figure 1C) were purchased from Sigma-Aldrich (St. Louis, MO, USA). Methanol was purchased from Honeywell Burdick and Jackson (Muskegon, MI, USA). Acetic acid and triethylamine were from Sigma-Aldrich (St. Louis, MO, USA). Water was purified using the aquaMAX™, ultra-pure water purification system (YL Instruments, Anyang, Korea). All other chemicals and solvents were of reagent or HPLC grade and used without further purification.

### 2.2. UHPLC System and Chromatographic Condition

The analysis was performed using the Agilent 1290 Infinity II UHPLC system (Agilent Technologies, Santa Clara, CA, USA), equipped with an auto-sampler (G7167B), a flexible pump (G7104A), a Multicolumn Thermostat (MCT) (G7116B), and a DAD detector (G7117A). A Synergi™ 4 µm polar, Reversed-Phase (RP) 80A column (150 × 2.0 mm, Phenomenex, Torrance, CA, USA) was used for separation. Isocratic elution was employed with a mobile phase consisting of 1% acetic acid, pH 5.5 (containing 0.01% triethylamine), and methanol (76:24, *v*/*v*) at a flow rate 0.2 mL/min. Before use, the mobile phase was adjusted to pH 5.5, filtered, and degassed. The injection volume was set at 2 µL, and the DAD detector to 280 nm. The column and autosampler tray were maintained at 25 °C and 4 °C, respectively.

### 2.3. Preparation of Calibration Standards and Quality Control (QC) Samples

Stock solutions of PA, NAPA, and NPPA (IS) were prepared separately at 1 mg/mL by dissolving accurately-weighed amounts of the compound in Double-Distilled Water (DDW). A series of working standard solutions of each compound were prepared by serial dilution of the respective stock solutions with DDW. A working internal standard solution of 200 ng/mL was prepared by diluting the IS stock solution in methanol. Calibration curve standard samples of PA and NAPA were prepared by spiking 90 µL of drug-free rat plasma with 10 µL of working standard solutions to give final concentrations of 10, 20, 50, 100, 200, 500, 1000, 2000, 5000, 10,000, and 100,000 ng/mL for PA, and 10, 20, 50, 100, 200, 500, 1000, 2000, 5000, and 10,000 ng/mL for NAPA. QC samples were prepared in the same way to final concentrations of 20 (Lower Limit of Quantification (LLOQ)), 60 (low QC), 8000 (mid QC), and 80,000 ng/mL (high QC) for PA, and 20 (LLOQ), 60 (low QC), 800 (mid QC), and 8000 ng/mL (high QC) for NAPA. All stock and working solutions were stored at −20 °C until required for analysis.

### 2.4. Sample Preparation

A simple protein-precipitation method was used for the analysis. Plasma samples (100 µL) were transferred into separate Eppendorf tubes and an aliquot of 200 µL of IS (200 ng/mL of NPPA in methanol) was added. After vortex-mixing for 1 min and centrifugation at 15,000 rpm for 15 min at 4 °C, 2 µL aliquots of the methanolic supernatants were injected into the UHPLC system.

### 2.5. Method Validation

The devised method was validated for selectivity, sensitivity, linearity, accuracy, precision, recovery, and stability. The selectivities for PA and NAPA were evaluated to determine possible interference by endogenous substances. Blank plasma samples from six randomly-selected Sprague-Dawley (SD) rats were used, and the UHPLC chromatograms of blank plasma, blank plasma spiked with PA (100 ng/mL), blank plasma spiked with NAPA (100 ng/mL), and blank plasma spiked with IS (200 ng/mL) were compared. Linearity was assessed by plotting the peak area ratios of each analyte to IS versus the nominal concentrations ranging from 20–100,000 and 20–10,000 ng/mL for PA and NAPA, respectively. Least squares regression with a weighting factor of 1/x was used to construct five calibration curves and to determine the correlation coefficient. Intra-day and inter-day precision and accuracy for PA and NAPA were evaluated by analyzing six replicates of QC samples (20, 60, 8000, and 80,000 ng/mL for PA and 20, 60, 800, and 8000 ng/mL for NAPA) over 5 consecutive days. Accuracies were calculated as mean ratios of observed and nominal concentrations. The precision was defined as Relative Standard Deviation (RSD). Six sets of QC samples were prepared on five different days, and each set of samples was analyzed within 24 h. Limits of Detection (LODs) and LLOQs for PA and NAPA were determined by visual evaluation and using Signal-to-Noise ratios (S/N) of 3:1 and 5:1, respectively. The acceptance criteria for precision and accuracy at LLOQ are within 20% RSD for precision and between 80% and 120% for accuracy.

The percentage recoveries of PA and NAPA in plasma after deproteinization with methanol were calculated. UHPLC peak areas of PA and NAPA in plasma after deproteinization with methanol were compared with those obtained from four nominal concentrations in methanol. Percentage IS recovery was determined at a concentration of 200 ng/mL.

The stabilities of PA, NAPA, and IS were evaluated under different conditions. To determine the stabilities of stock solutions of analytes and the IS, three replicate stock solutions (100 ng/mL for PA and NAPA, and 200 ng/mL for IS) were analyzed and peak areas were compared with a stock solution after storage for 6 h at room temperature and 4 weeks at −20 °C. To determine the stabilities of analytes and IS in rat plasma, short-term stability, long-term stability, freeze-thaw stability, and autosampler stability were assessed at four concentrations of PA and NAPA (20, 60, 8000, and 80,000 ng/mL for PA and 20, 60, 800, and 8000 ng/mL for NAPA), with three replicates for each concentration. The short-term stability was assessed by allowing QC samples to stand at room temperature for 4 h prior to analysis; long-term stability by storing QC samples at −20 °C for 4 weeks; freeze-thaw stability cycle by subjecting QC samples to three cycles of freezing at −20 °C for 24 h; and thawing at room temperature. In addition, the analysis was repeated after 24 h to determine auto-sampler stability at 4 °C.

### 2.6. Application to Pharmacokinetic Studies of Procainamide HCl

The animal experiment was performed in accordance with the Guidelines for Animal Care and Use issued by Gachon University. Experimental protocols involving the animals used in this study were reviewed and approved by the Animal Care and Use Committee of the Gachon University (#GIACUC-R2017011, approval date on 25th May 2017). Sprague-Dawley (SD) rats (7–8 weeks old, 220–280 g) were purchased from Nara Biotech (Pyeongtaek, Korea). Food and water were freely provided, and animals were allowed a week to adjust to the laboratory environment before commencing the experiment and maintained under a 12:12 h light/dark cycle.

To evaluate the relevance of the analysis method, an Intravenous (IV) pharmacokinetic study of PA was performed in SD rats. Rats were anesthetized (with a mixture of Zoletil and Rompun) and then a femoral vein and artery were cannulated for drug administration and blood sample collection, respectively [29]. PA HCl in saline (10 mg/kg) was then administered (*n* = 5) via the cannulated femoral vein. Blood was then collected at 0 (blank), 1, 5, 15, 30, 60, 120, 180, 240, 360, and 480 min after drug administration. After each blood sampling, the volume of blood collected was replaced with an equal volume of saline to compensate for blood loss. Collected blood was immediately centrifuged at 14,000 rpm for 15 min at 4 °C, and plasma was then separated and stored at −20 °C until analysis.

The plasma concentration–time profiles of PA and NAPA were plotted and analyzed using the non-compartmental method using WinNonlin (Ver. 5.0.1) [29]. For PA, the Area Under the plasma Concentration–time curve (AUC) was calculated using the linear trapezoidal method. AUC_last_ (from time zero to last time point), AUC_inf_ (from time zero to infinity), total body Clearance (CL), Volume of distribution at steady state (V_ss_), Mean Residence Time (MRT), and elimination half-life (t_1/2_), of PA were determined individually. Similarly, the pharmacokinetic parameters of the major metabolite, NAPA, were obtained including peak Concentration (C_max_) and Time to reach C_max_ (T_max_). In addition, the AUC ratio of PA and NAPA (AUC_NAPA_/AUC_PA_) was calculated using AUC_inf_ values for each rat.

## 3. Results and Discussion

### 3.1. Optimization of Chromatographic Analysis

To develop a simple method suitable for the simultaneous determination of PA and NAPA, chromatographic conditions, such as, eluent, column, and column conditions, were optimized. The mobile phase used had a methanol content that differed slightly from that used in a previously-reported method [20]. Namely, a slight increase in methanol content in the mobile phase improved the peak shapes and shortened the total run time. A polar-RP column was found to provide suitable retention times and better peak shapes than other columns (e.g., C-8 and C-18 columns). For example, tested C-8 and C-18 columns generated peak tailing, with a poor quantitation limit, or required a long retention time of IS (i.e., more than 30 min).

To obtain the highest recovery using a straightforward method, we selected deproteinization by methanol over liquid-liquid or solid-phase extraction. The deproteinization method was further optimized in terms of organic solvent. We tested acetonitrile and methanol, and subsequently selected methanol because it provided better peak shapes and sensitivity.

### 3.2. Selectivity

**Figure 2:**
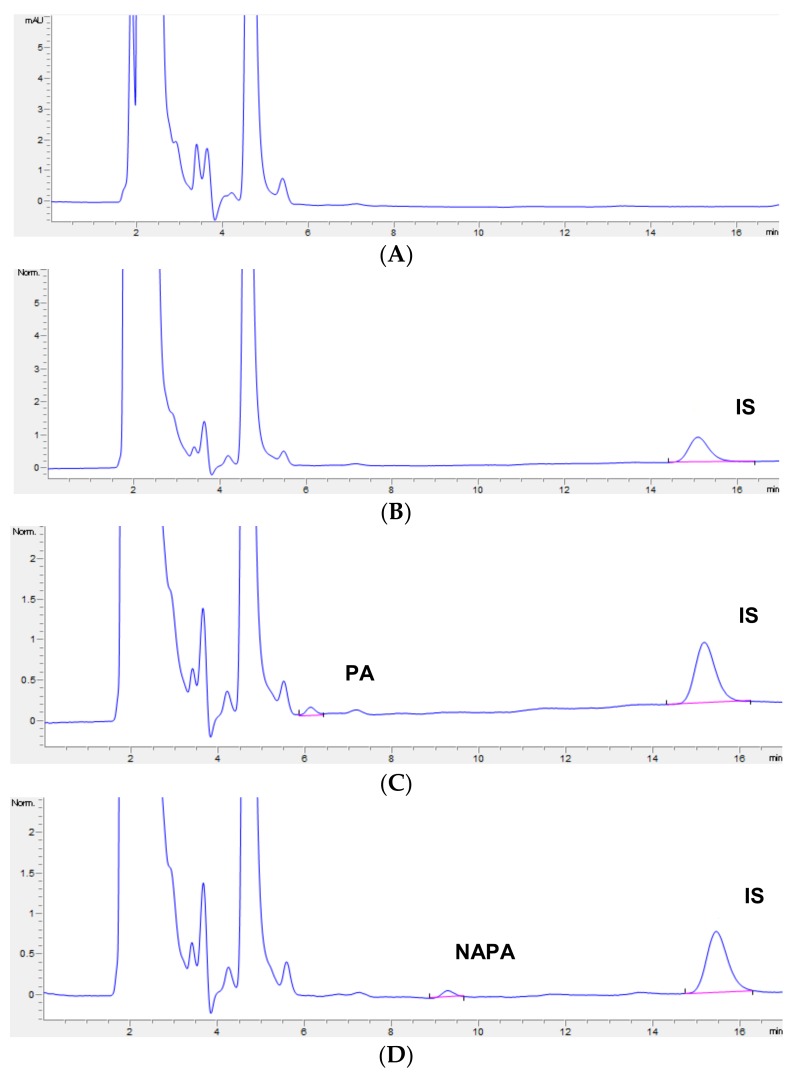

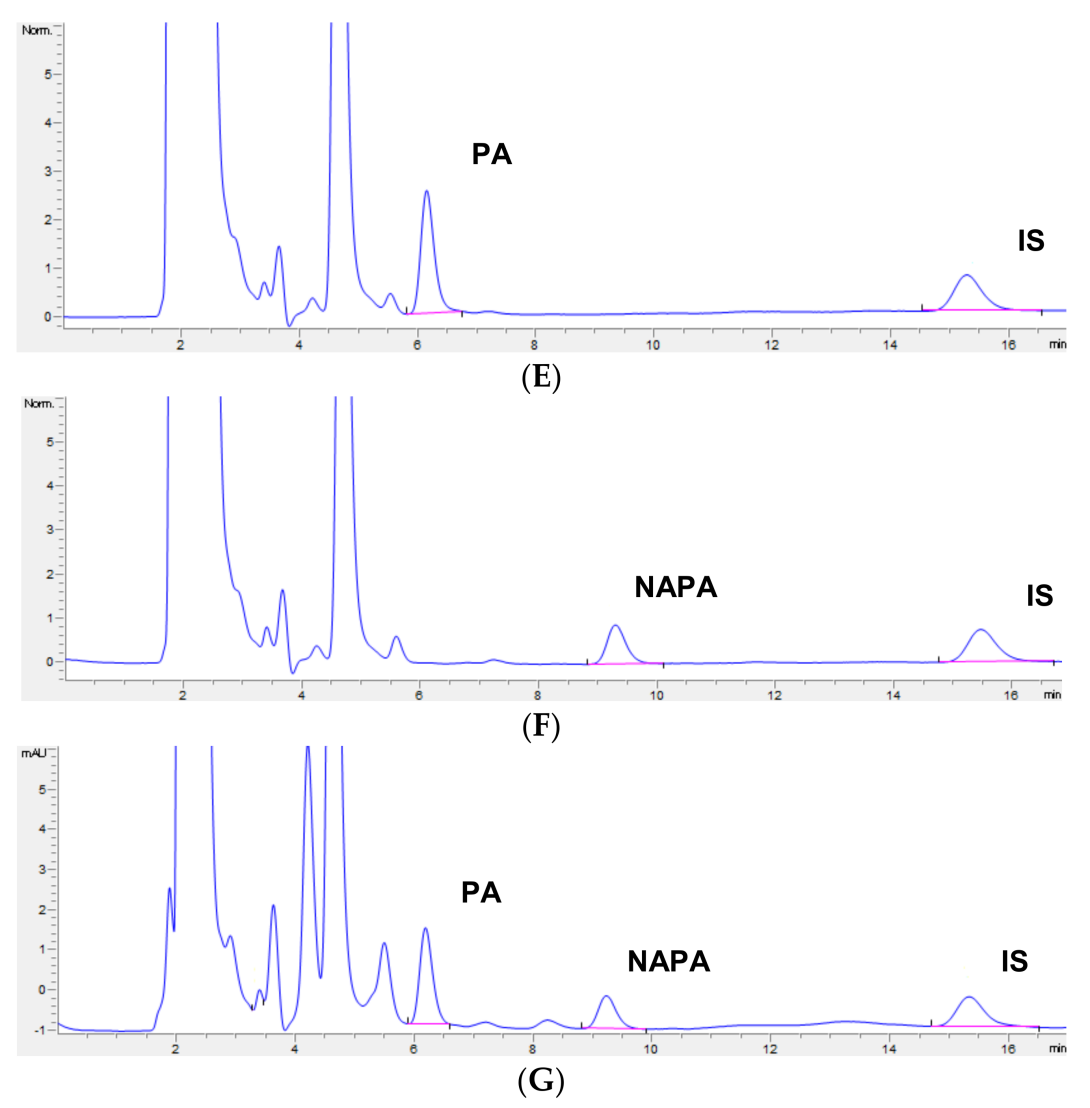
Chromatograms of (**A**) blank rat plasma; (**B**) plasma spiked with IS (200 ng/mL); (**C**) plasma spiked with procainamide (20 ng/mL, lower limit of quantification (LLOQ)) and IS (200 ng/mL); (**D**) plasma spiked with *N*-acetylprocainamide (20 ng/mL, LLOQ) and IS (200 ng/mL); (**E**) plasma spiked with procainamide (1000 ng/mL) and IS (200 ng/mL); (**F**) plasma spiked with *N*-acetylprocainamide (500 ng/mL) and IS (200 ng/mL) and (**G**) 30 min after intravenous administration of procainamide hydrochloride (10 mg/kg). PA: procainamide, NAPA: N-acetylprocaiamide, IS: internal standard.

UHPLC chromatograms of blank rat plasma samples verified the absence of interference at the retention times of PA, NAPA, and IS, which were 6.8, 9.9, and 15.6 min, respectively. Figure 2 shows representative chromatograms of blank plasma (Figure 2A), plasma spiked with IS (Figure 2B), plasma spiked with PA and IS (Figure 2C,E), and plasma spiked with NAPA and IS (Figure 2D,F).

### 3.3. Linearity

The calibration curve (*n* = 5) for PA was linear over the concentration range 20–100,000 ng/mL, and that for NAPA was linear over the range 20–10,000 ng/mL. Least squares regression with a weighting factor of 1/x was used to determine the calibration curves of PA and NAPA. The calibration equation for PA was y = (0.00198 ± 0.00007) x + (0.00804 ± 0.00391) with a coefficient of determination (*r*^2^) of 0.9995 ± 0.0004, the calibration equation for NAPA was y = (0.00262 ± 0.00015) x + (0.03151 ± 0.01200) with a coefficient determination (*r*^2^) = 0.9984 ± 0.0012. The results indicate that UHPLC-DAD response for both analytes was directly proportional to analyte concentration in plasma, and that assays were linear and reliable.

### 3.4. Precision and Accuracy

To determine intra- and inter-day accuracies and precisions, QC samples of PA and NAPA were prepared at concentrations of 20, 60, 8000, and 80,000 ng/mL and 20, 60, 800, and 8000 ng/mL, respectively. Six replicates were analyzed at each concentration. A summary of intra- and inter-day accuracies and precisions of PA and NAPA is provided in Table 1. Intra-day accuracies for PA ranged from 98.6 to 110.9% and RSDs from 2.0 to 7.5%, and inter-day accuracies ranged from 97.7 to 100.1% and RSDs from 5.2 to 10.5%. Similarly, intra-day accuracies for NAPA ranged from 99.7 to 109.2% and RSDs from 1.0 to 6.1%, and inter-day accuracies ranged from 100.8 to 106.3% and RSDs from 4.6 to 10.5%. Hence, intra- and inter-day accuracies and precisions for both analytes were within the ranges of standard FDA guidelines [30].

### 3.5. Sensitivity

Initially, LODs and LLOQs of PA and NAPA were determined by visual evaluation and found to be 10 and 20 ng/mL, respectively. LOD and LLOQ values were also determined using S/N ratios with acceptability of ≥3 for LOD and ≥5 for LLOQ. The S/N ratios for the LOD and LLOQ of PA were 3.7 and 6.7, respectively, and for NAPA were 3.5 and 5.9, respectively. Further, the LLOQ values for both analytes were confirmed to meet specified precision and accuracy values. The LLOQs for PA and NAPA were confirmed at 20 ng/mL, with accuracies less than 113.7% and 117.3% for PA and NAPA, respectively, and percent RSD of 10.5% for both PA and NAPA. The representative UHPLC chromatograms at LLOQ of PA and NAPA are shown in Figure 2C,D. Compared to LLOQs from previous literatures, it is indicated that our LLOQs for PA and NAPA using UHPLC-DAD are highly sensitive, without condensation in rat plasma. Although a previously-reported HPLC method with LLE provided better sensitivity for both PA and NAPA, this method is unlikely accessible for the preclinical pharmacokinetic studies due to the large sample volume required (i.e., 500 µL) [20].

### 3.6. Recovery

PA recovery was determined at concentrations of 20, 60, 8000, and 80,000 ng/mL and NAPA recovery at concentrations of 20, 60, 800, and 8000 ng/mL. IS recovery was determined at 200 ng/mL. Mean PA recovery values ranged from 95.6 to 103.3% and RSDs from 0.4 to 8.3%, while mean NAPA recovery values ranged from 93.7 to 100.0% and RSDs from 0.4 to 10.8%. Thus, recoveries for both analytes were near 100% and reproducible.

### 3.7. Stability

To determine the stock solution stabilities of PA, NAPA, and IS (100 ng/mL for PA and NAPA, and 200 ng/mL for IS), analytes were analyzed in triplicate after standing at room temperature for 6 h or at −20 °C for 4 weeks. As compared with fresh stock solutions, the determined stability values (%) of PA, NAPA, and IS were 103.3, 99.8, and 101.3%, respectively, after standing at room temperature for 6 h, and 104.6, 103.4, and 102.0%, respectively, after storage for 4 weeks at −20 °C. The stabilities of PA and NAPA in rat plasma were determined at four concentrations of PA and NAPA (20, 60, 8000, and 80,000 ng/mL for PA and 20, 60, 800, and 8000 ng/mL for NAPA) in triplicate. As compared with freshly-prepared QC samples, the stability (%) of PA in plasma QC samples allowed to stand at room temperature for 4 h was 91.6–108.1%, and that of samples stored at −20 °C for 4 weeks was 91.6–107.8 %. The stability (%) of PA after three freeze-thaw cycles, and after storage at 4 °C in an autosampler for 24 h, were 95.6–103.6% and 94.4–104.6%, respectively. Similarly, short-term, long-term, freeze-thaw, and autosampler stability ranges of NAPA were 95.3–102.0%, 92.4–102.6%, 96.3–102.7%, and 98.3–102.7%, respectively. The results of stability studies for PA and NAPA are listed in Table 2. These results show that PA and NAPA were stable under these storage and processing conditions.

### 3.8. Application to Pharmacokinetic Studies of Procainamide HCl

**Figure 3:**
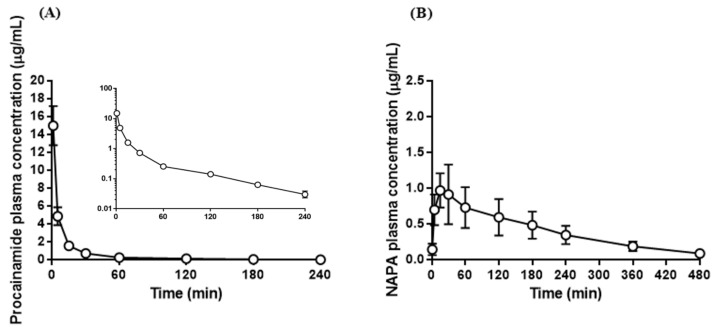
Application of the developed method to a pharmacokinetic study of procainamide; Plasma concentration-time profile of (**A**) procainamide and (**B**) *N*-acetylprocainamide after intravenous administration of procainamide hydrochloride (10 mg/kg) in rats (mean ± SD, *n* = 5).

The validated analytical method was applied to a pharmacokinetic study of PA HCl in SD rats that were administered a single dose of 10 mg/kg IV. Representative chromatograms from plasma samples collected 30 min after IV administration are shown in Figure 2G. PA concentrations in plasma were measurable for up to 4 h after injection, whereas NAPA was measurable for up to 8 h, as shown in Figure 3. The pharmacokinetic parameters determined were as follows: t_1/2_, AUC_last_, AUC_inf_, MRT, CL, V_ss_, C_max_, and T_max_. The results are summarized in Table 3. The AUC_inf_, CL, V_ss_, and t_1/2_ of PA were 136 ± 12.1 μg∙min/mL, 73.8 ± 6.51 mL/min/kg, 2070 ± 316 mL/kg, and 52.4 ± 2.20 min, respectively. The AUC_inf_ and t_1/2_ of NAPA were 177 ± 29.5 and 131 ± 31.7, respectively. The mean AUC_NAPA_/AUC_PA_ ratio (based on AUC_inf_ values) was 1.30 ± 0.191. The pharmacokinetic parameters of PA were comparable to those reported by previous studies [31,32,33]. Namely, CL, V_ss_, and t_1/2_ were 61.94–119.6 mL/min/kg, 3720–4860 mL/kg, and 47.4–55 min, respectively, after IV administration of PA (50 mg/kg) in rats [31,32,33]. The pharmacokinetic parameters of NAPA were also similar to a previous report [32]. The terminal half-life of NAPA after IV administration of PA (50 mg/kg) in rats was 104 min [32]. Collectively, these observations indicate that the devised UHPLC-DAD method can be employed to assay plasma samples and perform pharmacokinetic studies on PA and NAPA.

## 4. Conclusions

In the current study, we devised a UHPLC-DAD-based method for the simultaneous quantification of PA (a commonly-used antiarrhythmic agent) and its major metabolite, NAPA. The developed method has advantages over previously-reported HPLC methods. Namely, it requires only a single protein precipitation step to prepare plasma samples, and is straightforward, rapid, and cheap. In addition, it requires a relatively small volume (100 µL) of plasma for pharmacokinetic studies, which invariably involve the testing of large numbers of samples, and the small injection volume (2 µL) used minimizes carryover. The method exhibits good linearity and precision across the concentration ranges 20–100,000 ng/mL for PA and 20–10,000 ng/mL for NAPA. In conclusion, the technique used could be applied to determine concentrations of PA and NAPA simultaneously in preclinical studies.

## Figures and Tables

**Table 1:** Accuracy and precision of procainamide and *N*-acetylprocainamide in plasma.

Analytes	Nominal Concentration (ng/mL)	Intra-day (*n* = 6)			Inter-day (*n* = 30)		
		Measured Concentration (ng/mL)	Precision (RSD %)	Accuracy (%)	Measured Concentration (ng/mL)	Precision (RSD %)	Accuracy (%)
PA	20	22.1	2.0	110.3	19.5	10.5	97.7
	60	60.5	7.5	110.9	59.9	7.1	99.8
	8000	7890	4.2	98.6	8011	5.2	100.1
	80,000	79,079	4.0	98.9	78,994	6.0	98.7
NAPA	20	20.0	6.1	99.7	20.9	10.5	104.3
	60	62.1	3.3	103.4	60.5	9.1	100.8
	8000	873.2	1.0	109.2	850.1	4.6	106.3
	80,000	8031	5.1	100.4	8238	6.1	103.0

PA: procainamide; NAPA: *N*-acetylprocainamide; RSD: relative standard deviation.

**Table 2:** Stability of procainamide and *N*-acetylprocainamide in plasma ^1^.

Concentration (ng/mL)	Stability (%)	
	PA	NAPA
Freeze-thaw stability (3 cycles)		
20	103.6 ± 7.9	97.3 ± 6.5
60	98.9 ± 1.5	96.3 ± 2.0
8000	99.8 ± 1.0	102.7 ± 1.8
80,000	95.6 ± 0.8	100.3 ± 2.2
Auto-sampler stability (24 h at 4 °C)		
20	104.6 ± 6.0	102.7 ± 0.9
60	103.1 ± 2.2	98.3 ± 1.2
8000	103.4 ± 2.2	101.1 ± 3.0
80,000	94.4 ± 1.5	100.2 ± 1.7
Short-term stability (4 h at room temperature)		
20	108.1 ± 6.7	97.2 ± 9.4
60	103.5 ± 11.1	99.0 ± 1.6
8000	104.0 ± 2.3	102.0 ± 2.7
80,000	91.6 ± 4.6	95.3 ± 2.4
Long-term stability (4 week at −20 °C)		
20	107.8 ± 1.1	92.4 ± 2.5
60	106.1 ± 12.1	102.6 ± 2.5
8000	101.5 ± 2.4	96.4 ± 1.1
80,000	91.6 ± 4.6	97.3 ± 3.2

^1^ Results are presented as Mean ± SD (*n* = 3); PA: procainamide; NAPA: *N*-acetylprocainamide.

**Table 3:** Pharmacokinetic parameters of procainamide and its metabolite, *N*-acetylprocainamide after intravenous administration of procainamide hydrochloride (10 mg/kg) in rats (mean ± SD, *n* = 5).

Parameter	Procainamide	*N*-acetylprocainamide
AUC_last_ (µg·min/mL)	134 ± 12.5	156 ± 36.8
AUC_inf_ (µg·min/mL)	136 ± 12.1	177 ± 29.6
t_1/2_ (min)	52.4 ± 2.20	131 ± 31.7
MRT (min)	28.5 ± 2.99	181 ± 38.3
V_ss_ (mL/kg)	2070 ± 316	-
CL (mL/min/kg)	73.8 ± 6.51	-
C_max_ (µg/mL)	-	0.949 ± 0.124
T_max_ (min)	-	21.0 ± 8.22
AUC_NAPA_/AUC_PA_ ratio	1.30 ± 0.191	

## Supplementary Materials

The following are available online at http://www.mdpi.com/1999-4923/10/2/41/s1, Table S1: Summary of HPLC bioanalytical method for simultaneous determination of procainamide and *N*-acetylprocainamide in the previous literatures.
