# Supplementary material for: Simultaneous Determination of Procainamide and N-acetylprocainamide in Rat Plasma by Ultra-High-Pressure Liquid Chromatography Coupled with a Diode Array Detector and Its Application to a Pharmacokinetic Study in Rats
Source: Pharmaceutics. 2018 Mar 30;10(2):41. doi: 10.3390/pharmaceutics10020041 (PMC6027534; doi:10.3390/pharmaceutics10020041)
Supplement: Supplementary file 1 [file pharmaceutics-10-00041-s001.pdf]

Supplementary Materials: Simultaneous Determination of Procainamide and *N*-acetylprocainamide in Rat Plasma by Ultra-High-Pressure Liquid Chromatography Coupled with a Diode Array Detector and Its Application to a Pharmacokinetic Study in Rats

Anusha Balla, Kwan Hyung Cho, Yu Chul Kim and Han-Joo Maeng

Table 1. Summary of HPLC bioanalytical method for simultaneous determination of procainamide and *N*-acetylprocainamide in the previous literatures.

Sample preparation (extraction solvent)	Sample source	Required sample volume (µL)	Required injection volume (µL)	LLOQ (ng/mL)		Linear range (ng/mL)	Refs
				PA	NAPA		
LLE (methylene chloride)	Human plasma	2000	100	100	100	100–800	[16]
LLE (10% n-propanol in chloroform)	Human plasma	500	100	1000	1000	1000–15,000	[17]
LLE (methylene chloride and 2-propyl alcohol)	Human serum and urine	100	50	50	50	78.1–10,000	[18]
LLE (ethylacetate) Deproteinization (ACN)	Human, dog, and rat plasma	100	50	50	50	50-10,000	[19]
LLE (methylene chloride)	Human plasma	500	45	4	4	4–100 (low) 200–2500 (high)	[20]
LLE (chloroform)	Human serum	100	20	2500	2500	2500–15,000	[21]
LLE (methylene chloride)	Human blood	500	100	100	250	NA	[22]
LLE (methylene chloride)	Human plasma	500	10	500	500	500–15,000	[23]
LLE (methylene chloride)	Human plasma	200	25	NA	NA	NA	[24]
LLE (20% 1-butanol, 20% chloroform, and 60% hexane)	Human plasma	100	200	2000	2000	2000–20,000	[25]
LLE (49% methylene chloride, 49% hexane and 2% isoamyl alcohol)	Human plasma	2500	75	220	110	220–14,000 (PA) 110–14,000 (NAPA)	[26]

LLE: liquid-liquid extraction; PA: procainamide; NAPA: *N*-acetylprocainamide; LLOQ: lower limit of quantification; NA: not available.
